# The efficacy of the leg swing and quadriceps strengthening exercises versus platelet-rich plasma and hyaluronic acid combination therapy for knee osteoarthritis: A retrospective comparative study

**DOI:** 10.1097/MD.0000000000035238

**Published:** 2023-09-15

**Authors:** Cong Ma, Xuejing Li, Ying Pan, Hua Tian, Zhongzheng Wang, Xiaoyang Zhang, Xiaozuo Zheng, Guoqiang Liu, Kunfeng Duan, Suhui Qie

**Affiliations:** a Department of Pharmacy, Third Hospital of Hebei Medical University, Shijiazhuang, Hebei, PR China; b The Second Operating Room, Third Hospital of Hebei Medical University, Shijiazhuang, Hebei, PR China; c Department of Orthopaedic Surgery, Third Hospital of Hebei Medical University, Shijiazhuang, Hebei, PR China.

**Keywords:** exercise, hyaluronic acid, knee osteoarthritis, platelet-rich plasma

## Abstract

The aim of this was to investigate the efficacy of physical exercise (leg swing and quadriceps strengthening exercises) versus platelet-rich plasma (PRP) and hyaluronic acid (HA) combination therapy. From January 2020 to August 2021, 106 patients with Kellgren–Lawrence Grade I–III knee osteoarthritis were divided into leg swing and quadriceps strengthening exercises (Group A) and intra-articular combination injections of PRP and HA (Group B) according to the treatment strategies. Patients in Group A received regular leg swing and quadriceps strengthening exercises for 3 months. Patients in Group B received 2 intra-articular combination injections of PRP (2 mL) and HA (2 mL) every 2 weeks. The primary outcome measures were the Visual Analogue Scale (VAS) and the Western Ontario and McMaster Universities (WOMAC) score. Secondary outcomes included single leg stance test and functional activity by 2-minute walk test and time up and go test. All outcomes were evaluated at baseline and again 1, 3, 6, and 12 months. The VAS and WOMAC scores were similar in both groups at 1 and 3 months after treatment (*P* > .05); however, Group A patients had significantly superior VAS and WOMAC scores than Group B patients at 6 and 12 months after treatment. For the single leg stance test, 2-minute walk test, and time up and go test, Group A patients were significantly superior to Group B throughout follow-up (*P* < .001). The leg swing and quadriceps strengthening exercises resulted in a significantly better clinical outcomes than the combined PRP and HA therapy, with a sustained lower pain score and improved quality of life, balance ability, and functional activity within 12 months.

## 1. Introduction

Knee osteoarthritis (KOA) is one of the most prevalent musculoskeletal diseases and a leading cause of joint pain, stiffness, and increasing disability. It is mainly accompanied by articular cartilage injury, joint margin degradation, subchondral bone hyperplasia, cyst formation, muscular dystrophy, and weakness.^[1–3]^ Knee osteoarthritis (KOA) seriously affects the quality of life of middle-age and elderly patients and is a global public health issue.^[4]^ Patients with mild to moderate KOA (Grade II to Grade III, according to the Kellgren–Lawrence [K–L] classification) can often be managed with nonsurgical strategies. Currently, with the confirmation of the nephrotoxicity and side effects of oral non-steroidal anti-inflammatory drugs and corticosteroids, intra-articular platelet-rich plasma (PRP) and/or hyaluronic acid (HA) injections and physical exercise have become a potential alternative option for the treatment of KOA.^[5–7]^

PRP and HA, as commonly used drugs for intra-articular injection, have been proven to be effective treatment strategies for KOA.^[1,8]^ Autologous PRP is an autologous and multifunctional platelet concentrate from the blood that can release macrophages and growth factors after activation, stimulate the cartilage healing process, and improve the damage caused by joint disease.^[9,10]^ HA is a glycosaminoglycan that can lubricate articular surfaces, transport nutrients, repair degraded articular cartilage, and further delay joint degeneration of the knee.^[11,12]^ Several studies have demonstrated the efficacy of intra-articular PRP combined with HA injections in the treatment of KOA with promising clinical outcomes.^[1,6,8]^ The combination injections of PRP and HA seem to have a synergistic effect and can better improve the pain and functional status of patients than PRP or HA alone.^[13,14]^

Many documents illustrate that exercise therapy is widely used in the conservative management of KOA, and that moderate physical exercise can effectively improve pain, functional activities, quality of life, and psychological health in KOA patients.^[15,16]^ Currently, common physical exercise for patients with KOA includes swimming, walking, quadriceps strength training, leg swings, Tai Chi, cycling, yoga, or other forms of exercise.^[17]^ In clinical practice, the authors found that leg swing and quadriceps strengthening exercise for patients with KOA have better safety and economic benefits than other exercise methods, which can improve knee joint function of the knee, improve the health status of life, relieve pain, and delay knee cartilage degeneration. Therefore, the aim of this study was to investigate the efficacy of leg swing and quadriceps strengthening exercises versus the combination of PRP and HA injection in patients with KOA.

## 2. Materials and methods

### 2.1. Ethics approval and consent to participate

This study was designed as a retrospective study, and was performed following the ethical standards of the 1964 Declaration of Helsinki and later amendments. The study protocol was approved by the local ethics committee of our hospital (Approval No. W2021-039-1). The study had been retrospectively registered on Protocol Registration and Results System clinicaltrials.gov (ID: NCT05585216). Written informed consent was obtained after sufficient explanation was provided to all patients.

### 2.2. Study design and population

A total of 150 patients with painful KOA who received conservative treatment from the joint surgery outpatient department of our hospital between January 2020 and August 2021 were included. The inclusion criteria were as follows: (1) patients aged ≥ 45 years; (2) KOA diagnosed according to the American College of Rheumatology Classification Criteria^[18]^; (3) patients with a K–L grade for KOA of I–III; and (4) a history of symptoms for more than 3 months and patients with independent mobility. Exclusion criteria included the following: (1) lower limb axial deviation >5° (valgus and varus knees); (2) patients with diabetes mellitus, severe cardiovascular disease, immunosuppressive status, mental illness; (3) injections or other invasive treatments of the lower extremities were used in the previous 3 months; (4) patients who were willing to undergo exercise training under the medical supervision; (5) patients with previous fractures or bone tumors of the ipsilateral lower limb; (6) patients who received previous hip or knee joint surgery; (7) patients who had participated in standardized lower limb exercises strengthening within the previous 3 months; and (8) patients who did not complete the regular follow-up. For patients with bilateral KOA, the more severely affected side (according to K–L grade, pain intensity, and knee joint range of motion). If the K–L grade of the bilateral knees was the same, the right side was included in the study.

**Figure 1.**
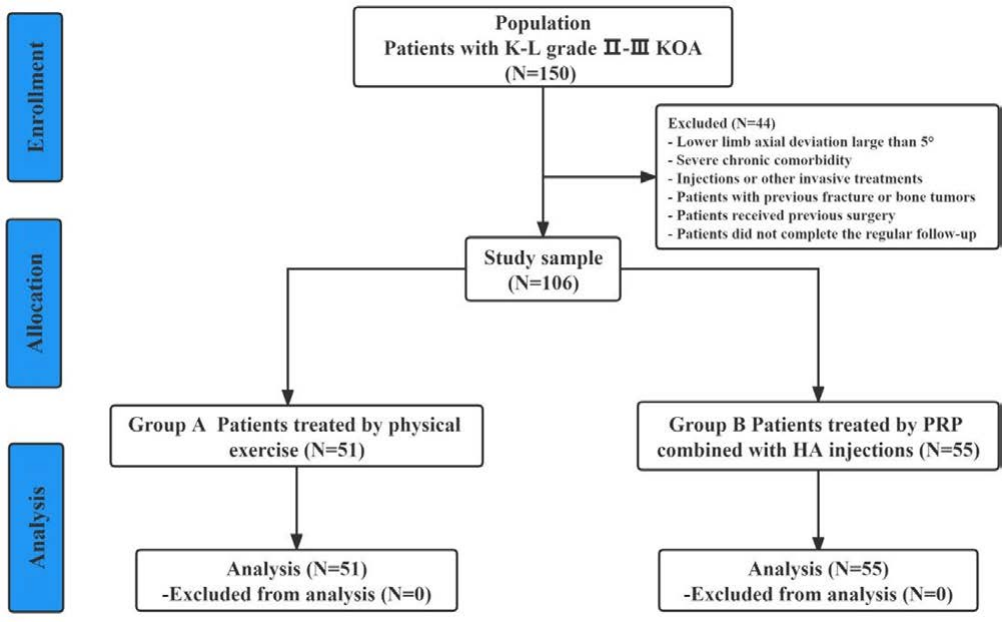
The flowchart of the patient screening process.

Finally, 106 (70.7%) patients with K–L Grade I–III KOA were enrolled, and 44 patients were lost to follow-up. According to the treatment strategies, 106 patients were divided into 2 groups: patients in Group A were treated with leg swing and quadriceps strengthening exercises (3 months); patients in Group B were treated with intra-articular combination injections of PRP (2 mL) and HA (2 mL) every 2 weeks. The affected knees were injected under ultrasound guidance at 30° of flexion. Injections were performed using a strictly sterile lateral mid-patellar approach. The HA was 2.0 mL (20 mg HA) of high molecular weight (2.4–3.6 million daltons) non-cross-linked HA extracted from bacterial cells (Eufflexa-Ferring 10 mg/mL HA), as shown in Figure 1.

**Figure 2.**
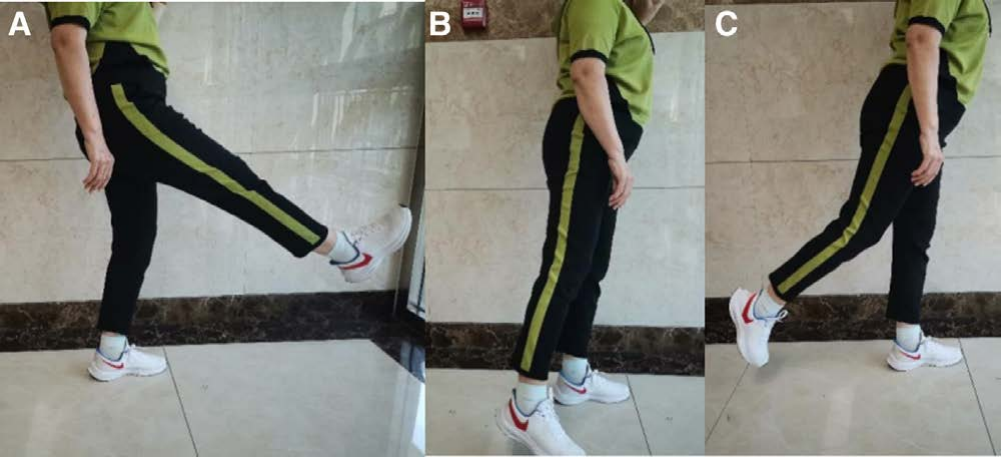
Patients swing leg exercise method. (A) Raising the affected leg to about 45° from the vertical line; (B) affected leg at the vertical line and slightly abducted; (C) swing the affected leg to the back.

**Figure 3.**
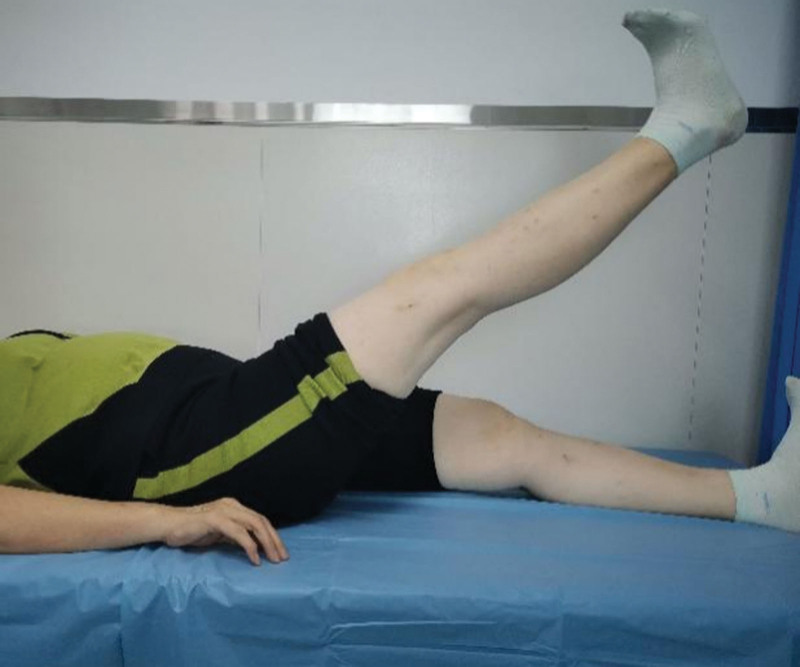
Quadriceps strengthening exercise method. Raising the affected leg until the heel was 25 to 30 cm away from the bed.

In this study, the physical exercises included leg swinging and quadriceps strengthening exercises. In the leg swing exercise, the unaffected leg is placed on the floor or the edge of a platform, allowing the affected leg to swing freely in the air. To prevent falling and maintain balance, one hand could be used to support the wall or railing. Patients could lift the limb to approximately 45° from the vertical line and then swing it to the back. Once patients were comfortable with the training, they were advised to swing the limb to approximately 60°. They were suggested to swing their limbs approximately 500 times per day (Fig. 2).^[19]^ For the quadriceps strengthening exercise, the ankle joint of the affected side was held in plantar flexion in a neutral position, the knee joint was extended to 0°, and then the leg was slowly raised until the heel was 25 to 30 cm away from the bed, held for 5 to 10 seconds and then slowly lowered to the supine position. They were required to raise their leg approximately 200 times per day (Fig. 3).^[20]^ These 2 physical exercises were interleaved in the patient’s spare time. Enrolled patients will be asked to perform this physical exercises at home every day for 3 months.

### 2.3. PRP preparation

Ten milliliters of blood was drawn from each patient’s antecubital vein. Blood samples were centrifuged at 1500 g (3400 rpm) for 15 minutes at room temperature, and 4 mL of PRP was obtained using a general-purpose centrifuge (Regen Lab). This was leukocyte-rich plasma and PRP according to the Dohan Enrenfest et al Classification.^[21]^ To initiate platelet coagulation activation, calcium chloride was added to the PRP solution prior to injection.

### 2.4. Outcome assessment

In the present study, the participants will be followed for at least 12 months. Before and after the treatment, observers measured pain severity, health status of life, balance ability, and functional activity. The primary outcome measures were the Visual Analogue Scale (VAS) and the Western Ontario and McMaster Universities (WOMAC) scores.^[3,19,20]^ Secondary outcomes included the single leg stance test (SLS) and functional activity using the 2-minute walk test (2MWT) and the time up and go test (TUGT).^[13,15]^ All outcomes were assessed at baseline and at 1, 3, 6, and 12 months.

Pain severity was evaluated by VAS score (range from 0 to 10, with 0 being no pain and 10 being worst possible pain). Quality of life was assessed by the WOMAC total score. The scale consisted of 8 indicators that determined the patient’s assessment of their physical performance. SLS is the time spent standing with the unaffected knee flexed and the affected leg supported, and is an important predictive parameter for assessing balance ability.^[13]^ The unaffected leg does not touch the supporting leg and remains balanced for as long as possible. Three trials were performed for each patient, and the best result of the 3 trials was recorded. Functional activity was evaluated using the 2MWT and TUGT at baseline and 1, 3, 6, and 12 months after treatment. The 2MWT is the distance the patients can walk in 2 minutes, with or without a walking aid. TUGT is the time patients need to get up from the same chair, walk as far as they can in a straight line for 3 meters, and then sit back down on the chair.^[15]^

### 2.5. Data analysis

Statistical analyses were performed using IBM SPSS Statistics Version 22.0 (SPSS Inc., Chicago, IL). The Kolmogorov–Smirnov test was used to check the normality of data distributions. Continuous variables are expressed as the mean and standard deviation, and categorical variables are expressed as numbers and percentages (%). The Mann–Whitney *U* test was used for continuous data, and the chi-squared test was used for categorical data. A *P*-value < 0.05 was considered statistically significant.

## 3. Results

### 3.1. Patient characteristics

Of the 150 participants, 106 patients (74 women and 32 men, mean age 63.85 ± 8.49 years) met the screening criteria and were divided into either the physical exercise group (n = 51) or the combined-injection group (n = 55). No patients were lost to follow-up during the study period. Demographic and clinical characteristics are shown in Table 1. According to the K–L classification, fifteen of 106 knees (14.15%) were documented as having Grade I, 34 as having Grade II, and 57 as having Grade III in both groups. The mean follow-up time of all patients was 16.77 ± 2.61 months. There were no statistically significant differences in demographic characteristics or baseline clinical data between the 2 groups (*P* > .05). No patients in either group experienced any adverse events during treatment.

**Table 1:** Demographic and baseline characteristics of the 2 treatment groups.

Variables	Group A (n = 51)	Group B (n = 55)	*P*-value
Age (years)	64.9 ± 8.7	62.7 ± 8.3	.238
Gender (female)	35 (68.6)	39 (70.9)	.835
BMI (kg/m^2^)	27.8 ± 3.8	28.6 ± 8.2	.590
Affected side (left)	23 (45.1)	22 (40.0)	.695
Smoking (yes)	7 (13.7)	9 (16.4)	.790
Alcoholism (yes)	4 (7.8)	7 (12.7)	.530
Living area			.846
Rural	21 (41.2)	24 (43.6)	
Urban	30 (58.8)	31 (56.4)	
Hypertension (yes)	14 (27.5)	16 (29.1)	.851
Follow-up time (months)	16.1 ± 2.5	16.5 ± 2.4	.399
K–L classification			.817
Grade I	8 (15.7)	7 (12.7)	.662
Grade II	15 (29.4)	19 (34.5)	.572
Grade III	28 (54.9)	29 (52.8)	.822

All values are expressed as mean ± standard deviation or numbers (%).

BMI = body mass index, K–L = Kellgren–Lawrence.

### 3.2. Main outcome measurements

As shown in Table 2, no significant differences in baseline VAS score, WOMAC scores total, SLS test, 2MWT, and TUGT test were found between the 2 groups. Both groups showed significant improvements in VAS pain and WOMAC score after treatment compared to before treatment. However, there was no significant difference between the 2 groups at 1 and 3 months after treatment. At 6 and 12 months after treatment, the VAS and WOMAC scores of Group A were significantly lower than those of Group B (*P* < .001). We then assessed the effects of the 2 treatment strategies on balance ability and functional activity in patients with KOA. Both groups showed significant improvements in knee strength at each follow-up period compared to baseline. The data also indicated a greater percentage of knee balance ability and functional activity between the 2 groups. At 1 month after treatment, patients who received PRP injection combined with leg swing and quadriceps strengthening exercise had a significant advantage in SLS, 2MWT, and TUGT (*P* < .05). There was also a tendency towards increased differences in SLS, 2MWT, and TUGT between groups at 3, 6, and 12 months after treatment was observed (*P* < .001).

**Table 2:** Primary and secondary outcomes at different follow-up in both treatment groups.

Variables	Group A (n = 51)	Group B (n = 55)	*P*-value
VAS score			
Baseline	7.09 ± 1.21	7.02 ± 1.07	.593
1 month	3.24 ± 0.89	3.43 ± 0.75	.363
3 months	3.06 ± 0.76	2.84 ± 0.88	.205
6 months	2.18 ± 0.82	4.27 ± 0.95	<.001
12 months	2.43 ± 0.51	5.15 ± 1.03	<.001
WOMAC score total			
Baseline	50.20 ± 1.67	50.49 ± 2.15	.530
1 month	30.16 ± 1.71	31.00 ± 2.24	.065
3 months	26.00 ± 1.96	25.40 ± 1.81	.124
6 months	24.73 ± 1.70	31.00 ± 2.07	<.001
12 months	28.51 ± 2.63	45.36 ± 3.60	<.001
SLS test (s)			
Baseline	28.53 ± 10.18	29.95 ± 9.31	.456
1 month	34.51 ± 10.51	31.91 ± 11.01	.043
3 months	51.35 ± 10.08	39.62 ± 10.65	<.001
6 months	57.14 ± 12.49	42.09 ± 10.93	<.001
12 months	56.25 ± 9.46	38.96 ± 10.41	<.001
2MWT (m)			
Baseline	110.55 ± 26.95	108.47 ± 28.08	.699
1 month	123.78 ± 17.59	115.70 ± 21.68	.038
3 months	135.10 ± 15.18	120.41 ± 19.03	<.001
6 months	139.90 ± 16.32	125.18 ± 20.25	<.001
12 months	133.55 ± 14.12	118.75 ± 21.53	<.001
TUGT (s)			
Baseline	12.43 ± 2.66	12.62 ± 2.88	.730
1 month	10.13 ± 2.23	11.24 ± 2.68	.046
3 months	8.55 ± 1.69	10.65 ± 2.29	<.001
6 months	8.03 ± 1.53	9.65 ± 2.17	<.001
12 months	8.47 ± 2.04	11.15 ± 2.74	<.001

All values are expressed as mean ± standard deviation or numbers (%).

2MWT = 2-minute walking test, SLS = single leg stance test, TUGT = time up and go test, VAS = Visual Analogue Scale, WOMAC = Western Ontario and McMaster Universities.

Notably, Group A showed significant improvements from baseline in pain intensity, quality of life, balance ability, and functional activity at each follow-up period, and the improvements were maintained after 6 months. In Group B, all primary and secondary observer measures improved significantly at 3 to 6 months, with maximal improvement was at 3 months, but he effects gradually diminished thereafter (Table 2).

## 4. Discussion

This study compared the efficacy of physical exercise versus intra-articular PRP combined with HA injection for patients with K–L Grade I–III KOA. We found that the leg swing and quadriceps strengthening exercise group and intra-articular PRP combined with HA injection both improved pain, quality of life, balance ability, and functional activity in patients with mild to moderate KOA. However, the improvements in the leg swing and quadriceps strengthening exercise group were more significant than those in the intra-articular PRP combined with HA injection group and were maintained after 6 months.

KOA is a degenerative disease with clinical manifestations including joint pain, stiffness, joint swelling, restricted movement, and joint deformities.^[22]^ Treatment of KOA includes nonsurgical treatment, such as weight loss, exercise, physical therapy, analgesic drugs, injections of HA and PRP, and surgical treatments.^[23]^ However, previous studies have shown that long-term use of non-steroidal anti-inflammatory drugs and analgesic drugs may cause nephrotoxicity and gastrointestinal side effects, as well as dependence, and lead to accelerated loss of knee function.^[3]^ Physical therapy can temporarily relieve pain and restore function, but it cannot maintain the effect for a long time.^[8]^

In recent years, the clinical application of intra-articular PRP and HA injection for the treatment of mild to moderate KOA has become increasingly frequent, and its therapeutic effect has been widely reported.^[1,6,8,13]^ In addition, some evidence suggests that physical exercise, as a safe and non-pharmacologic intervention for KOA, can also improve patient outcomes related to symptoms, quality of life, and functional activity.^[3,17,20]^ Exercise therapy can prevent muscle atrophy, treat and prevent pain, and improve lower limb motor function.^[24]^ The most commonly used physical exercises include quadriceps strengthening, leg swings, and hip abductor strength training. A number of studies have found that leg swing and quadriceps strengthening exercises may have a potentially positive effect on cartilage repair, significantly improving joint function, reducing humoral and cellular immune responses, and promoting angiogenesis, thereby improving histological parameters.^[25,26]^ In our study, leg swing and quadriceps strengthening exercises were shown to be an effective in the treatment of mild-to-moderate KOA and was superior to PRP combined with HA injection. These findings suggest that leg swing and quadriceps strengthening exercises may be a potential treatment option for patients with KOA in the future.

Clinically, VAS pain and WOMAC total score are commonly used to evaluate symptom relief and quality of life improvement in patients with KOA. Lana JF et al^[6]^ demonstrated that PRP combined with HA injection was effective in alleviating pain symptoms and WOMAC scores at 30 and 90 days after application. Consistent with our findings, intra-articular PRP combined with HA injection significantly improved the VAS score and WOMAC score total at 1 and 3 months compared with baseline data, but the improvement gradually diminished after reaching a peak at 3 months. Notably, there was no difference in the VAS and WOMAC scores of patients who received leg swing and quadriceps strengthening exercise compared with patients who received combined intra-articular injection of PRP and HA at 1 and 3 months after treatment. However, with longer follow-up, the advantage of exercise therapy in improving knee pain and quality of life gradually emerged (*P* < .001).

The latest KOA guidelines have given considerable attention to exercise rehabilitation and sports promotion strategies in disease management.^[7]^ Considering that the lower limb represents a whole chain of motion, studies investigating the effects of physical exercise on patients with KOA have mainly focused on quadriceps strengthening, hip joint peripheral muscle strengthening, and leg swinging. Some scholars have suggested that quadriceps strengthening exercises may be an effective way to prevent and treat KOA, which could reduce pain and restore stability and function.^[3,19,20,27]^ Meanwhile, some recent studies have found that leg swing exercise can reduce pain and improve the overall function of patients with KOA by strengthening the hip abductor muscle strength and reducing knee joint loading.^[19]^ In patients with KOA, pain and muscle wasting are associated with limited knee function. Chronic pain causes behavioral avoidance and increases muscle wasting. In addition, improvements in muscle strength, proprioception, and balance can have a major impact on lower limb mobility performance.^[15]^

The SLS, 2MWT, and TUGT tests are objective clinical tests of standing balance and functional activity. O’Reilly et al^[28]^ performed quadriceps strengthening exercises on patients with KOA for 6 months and found that joint pain was effectively relieved, VAS score was reduced by 22.5%, and the physical function score was significantly improved. Sun et al^[13]^ published a study evaluating SLS in patients with KOA after treatment with a combined injection of PRP and HA. The findings suggested that decreased lower limb muscle strength and proprioceptive deficits in KOA may affect effective and timely motor responses to maintain balance.^[29]^ In addition, Bokaeian et al^[15]^ used the 2MWT and TUGT to assess knee functional activity in patients with KOA after quadriceps strengthening training. The results showed that quadriceps strengthening training significantly improved the mean changes in knee functional activities, which was consistent with our findings. Our study showed that the SLS, 2MWT, and TUGT tests in patients who underwent leg swing and quadriceps strengthening exercise were significantly better than those in the combined injection group (*P* < .05) at 1, 3, 6, and 12 months after treatment. Our results suggest that the synergistic effect of leg swing and quadriceps strengthening exercise may be better than that of PRP combined with HA injection, but the specific mechanism remains unclear.

Several limitations to this study should be noted. First, this was a single center retrospective study, and the sample size was small. Only patients with K–L Grade I–III KOA were recruited. Secondly, the combined intra-articular injection volume was twice that of the single-injection groups. The larger volume may lead to overexpansion of the subcutaneous fascia, which may affect the observed results. Third, instead of systematically measuring balance ability and functional activity, we used indirect scoring tests to reflect the treatment effect. Finally, patients received different combination therapies, which could also affect the results.

## 5. Conclusions

The present study showed that leg swing and quadriceps strengthening exercises had a better clinical effect than PRP and HA combined therapy in patients with mild to moderate KOA. Leg swing and quadriceps strengthening exercise could significantly improve pain, quality of life, balance ability, and functional activity and maintain these improvements over 12 months. With exercise as part of a comprehensive treatment programme, we hope that our therapy will be the first choice for the patients with KOA.

## Author contributions

**Data curation:** Xuejing Li, Ying Pan, Guoqiang Liu.

**Formal analysis:** Xuejing Li, Xiaoyang Zhang, Kunfeng Duan.

**Methodology:** Cong Ma, Suhui Qie.

**Project administration:** Suhui Qie.

**Supervision:** Ying Pan, Hua Tian, Xiaozuo Zheng.

**Writing – original draft:** Cong Ma.

**Writing – review & editing:** Ying Pan, Zhongzheng Wang, Suhui Qie.
